# Harnessing omics to decode the mechanisms of symbiotic nitrogen fixation

**DOI:** 10.1007/s42994-025-00208-5

**Published:** 2025-03-26

**Authors:** Keyi Ye, Jianshu Zheng, Zhaonian Dong, Shuaishuai Wang, Sanwen Huang

**Affiliations:** 1 National Key Laboratory for Tropical Crop Breeding, Shenzhen Branch, Guangdong Laboratory of Lingnan Modern Agriculture, Genome Analysis Laboratory of the Ministry of Agriculture and Rural Affairs, Agricultural Genomics Institute at Shenzhen, Chinese Academy of Agricultural Sciences, Shenzhen, 518120 China; 2 National Key Laboratory for Tropical Crop Breeding, Chinese Academy of Tropical Agricultural Sciences, Haikou, 571101 China

**Keywords:** Symbiotic nitrogen fixation, Omics, Root nodule, Nodulation, Legumes

## Abstract

Symbiotic nitrogen fixation is predominantly observed in legumes, which form specialized structures termed nodules on their roots that contain symbiotic rhizobia. This mutualistic association provides reciprocal benefits: rhizobia convert atmospheric nitrogen into bioavailable forms, supplying essential nitrogen to their host plants, while obtaining reduced carbon in return. The increasing reliance on nitrogen fertilizers to satisfy escalating demands for food has prompted various approaches aimed at unravelling the mechanisms underlying symbiotic nodulation, seeking to transfer this capacity to non-nodulating crops. Transcriptome-based analyses have revealed that nodulation is a complex developmental program involving many genes. To comprehensively investigate this phenomenon, multiple omics technologies have been deployed and integrated, yielding exciting breakthroughs. In this review, we outline how omics have accelerated research in this area and discuss how advancements in technologies, such as artificial intelligence, could further deepen our understanding of nodulation.

**Supplementary Information:**

The online version contains supplementary material available at 10.1007/s42994-025-00208-5.

## Introduction

Symbiotic nitrogen fixation (SNF) is a biological program present in the nitrogen-fixing clade (NFC) of plant species, which comprises four orders: Fabales, Rosales, Fagales, and Cucurbitales. Nodulating plants within these orders can utilize nitrogen from the atmosphere by establishing a symbiotic relationship with nitrogen-fixing bacteria, which convert atmospheric nitrogen (N_2_) into ammonia. Within the Fabales, members of the Fabaceae, commonly known as legumes, establish symbiotic relationships with rhizobia. By contrast, nodulating plants from the three other orders form symbiotic associations with the actinobacterium *Frankia*, and are thus classified as actinorhizal plants, with members of the genus *Parasponia* within the Rosales being the only known plants with a rhizobial microsymbiont. Given our increasing reliance on nitrogen fertilizers driven by the escalating demand for food, SNF has garnered significant attention. Scientists are now focused on deciphering the mechanisms underlying SNF, aiming to transfer this capability to crops that cannot currently engage in symbiosis with rhizobia or actinobacteria.

Nodulation in legumes has been the focal point of most research due to the economic and agricultural significance of many leguminous species. The establishment of symbiotic relationships between legumes and rhizobia requires the proper initiation and progression of several crucial steps. In low-nitrogen environments, legumes release specific (iso) flavonoids into the rhizosphere to trigger the invasion of rhizobia into the plant. In response, rhizobia produce nodulation factors (Nod) that interact with plant receptor proteins, facilitating the establishment of the symbiotic relationship (Zhu and Yu 2024). Subsequently, plant tissues, primarily in the root cortex at the infection site, undergo dedifferentiation and mitosis to form nodule primordia. The rhizobia then enter nodule cells via endocytosis, developing into bacteroids with nitrogen-fixing capability. Bacteroids are encased by one layer of plant cell membrane, collectively forming organelles called symbiosomes. Through various transporters on the symbiosome membrane, plants supply rhizobia with essential nutrients for survival, including photosynthetic carbon and essential minerals, while rhizobia reciprocate by providing their host plant with nitrogen.

In the 1980s, scientists speculated that only dozens of genes were essential for SNF, based on the number of nodulation-related genes that had been discovered at that time (Roy et al. 2020). However, the revolution of genome sequencing and transcriptome studies in the early 2000s revealed that thousands of genes are actively expressed in nodules, suggesting their potential involvement in SNF (Benedito et al. 2008; Høgslund et al. 2009). Concurrently, forward and reverse genetic investigations have identified about 200 essential genes that contribute to SNF, encoding proteins with diverse functionalities, such as signal receptors, transporters, and transcription factors (Mergaert et al. 2020; Roy et al. 2020). Notably, some of these genes operate within interconnected pathways, collectively orchestrating nodulation progression and underscoring the complexity of SNF.

**Fig. 1:**
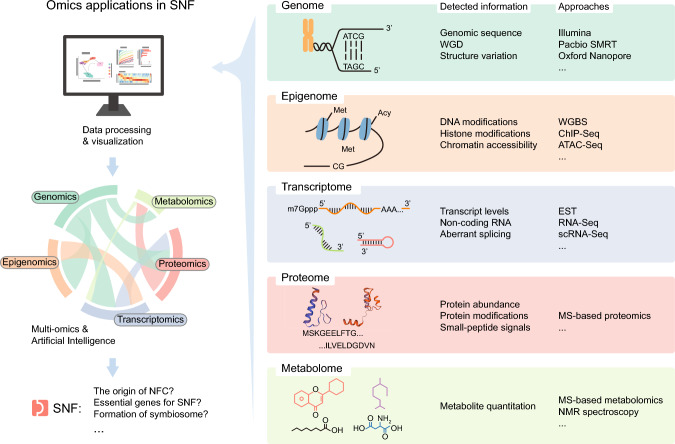
Applications of omics in symbiotic nitrogen fixation research. Omics approaches play a pivotal role in studies of SNF. These methodologies offer comprehensive insights into the genome, transcriptome, proteome, and beyond. By meticulously analyzing these diverse datasets, incorporating multi-dimensional data integration, and leveraging advanced artificial intelligence techniques, scientists are empowered to tackle and answer critical questions pertinent to SNF

To explore SNF from a global perspective, omics technologies have emerged as powerful tools, enabling researchers to query all genes, proteins, or metabolites associated with a given topic. By leveraging genomics, transcriptomics, epigenomics, proteomics, metabolomics, and other innovative techniques such as single-cell RNA sequencing (scRNA-seq) and spatial transcriptomics, scientists have begun to dissect SNF from broad evolutionary contexts to intricate gene functions (Fig. 1). This review summarizes these endeavors within the field of plant biology and offers visionary perspectives on how future advancements in omics approaches can help unravel the remaining enigmas surrounding SNF.

## Assembling and annotating the genomes of model legumes

The genome is a repository of information about the development, physiology, and evolution of an organism, serving as a crucial blueprint and framework for functional research and other omics investigations, such as transcriptomics and proteomics, which will be discussed in later sections (Lander et al. 2001; Venter et al. 2001). Model legumes such as *Lotus japonicus*, *Medicago truncatula*, and *Glycine max* have been extensively used in the study of SNF (Barker et al. 1990; Bolon et al. 2011; Handberg and Stougaard 1992). Among these, the *L. japonicus* genome was the first to be assembled, with a length of 315.1 Mb (Sato et al. 2008), estimated at the time to represent about 67% of the full genome (Ito et al. 2000). Although only 130 Mb was classified as high-quality and anchored to chromosomes, this accomplishment provided valuable data for investigating nodulation. For example, a survey of this initial genome sequence identified 12 genes encoding LysM receptor-like kinases (LysM-RLKs), including the critical Nod-factor receptors Nod Factor Receptor 1 (NFR1) and NFR5, which are essential for initiating nodule formation (Li et al. 2024b; Limpens et al. 2003; Madsen et al. 2003; Radutoiu et al. 2003). The remaining 10 LysM-RLKs were suggested to have potential roles in signaling pathways. Indeed, recent studies have highlighted LjNFRe, another such LysM-RLK, as a previously undescribed factor participating in nodule initiation (Murakami et al. 2018).

In 2010, the first high-quality genome for the soybean variety Williams 82 was reported (Schmutz et al. 2010), comprising 950 Mb of assembled sequences anchored to chromosomes, accounting for about 85% of the estimated genome (Arumuganathan and Earle 1991). Using the sequences of known nodulins (nodule-specific or nodule-enhanced genes) as queries against the newly released genome, a total of 28 nodulin homologs and 24 key regulatory genes were identified. The following year, the sequence of the *M. truncatula* genome was released (Young et al. 2011), comprising 367 Mb out of the ~ 465 Mb estimated *M. truncatula* full genome (Bennett and Leitch 2011). In particular, an analysis of the genome highlighted the retention of numerous paralogs stemming from a whole-genome duplication (WGD) event that occurred approximately 58 million years ago (Mya). Intriguingly, several of these paralogous genes have since likely undergone sub- or neo-functionalization in the context of nodulation. For example, *ERF REQUIRED FOR NODULATION 1* (*ERN1*), encoding a transcription factor essential for rhizobial infection (Middleton et al. 2007), has a paralog named *ERN2*, which participates in mycorrhizal colonization. This observation suggests that the WGD event that took place 58 Mya appears to have facilitated the sub-functionalization of an ancestral gene involved in both types of interactions, leading to the evolution of two related genes, each specialized in a single function.

The emergence of long-read sequencing technology significantly improved the quality of genome assembly (Garg et al. 2024; Li and Durbin 2024), further facilitating functional research and omics-based studies of SNF. For instance, researchers have employed long-read PacBio sequencing to enhance the genome assembly quality of *M. truncatula*, which resulted in a significant improvement in genome continuity, reducing the number of contigs from the previous 10,160 to merely 64 (Pecrix et al. 2018). The greatly improved genome has enabled a comprehensive analysis of transposable elements and their dynamics, as well as the identification of previously missed factors involved in nodule development. Particularly, researchers discovered symbiotic islands—genomic regions containing a large fraction of genes whose expression levels are upregulated in nodules or harboring genes that are exclusively expressed in the nodule differentiation zone. A subsequent study on *L. japonicus* using long-read PacBio sequencing has contributed valuable resources for research in legumes, and revealed the absence of symbiotic islands in *L. japonicus* (Kamal et al. 2020). Collectively, these findings underscore the profound impact of refined genome assemblies on enhancing our understanding of SNF.

## Genomic insights into the evolution of nodulating plants

Advancements in sequencing technology and the drop in associated costs have led to the successful sequencing of an increasing number of plant genomes. These extensive resources have been utilized for comparative genomics studies aimed at uncovering the shared traits of symbiotic nodulating plants and the characteristics that distinguish them from non-nodulating plant species, focusing on the conditions necessary for achieving SNF.

**Fig. 2:**
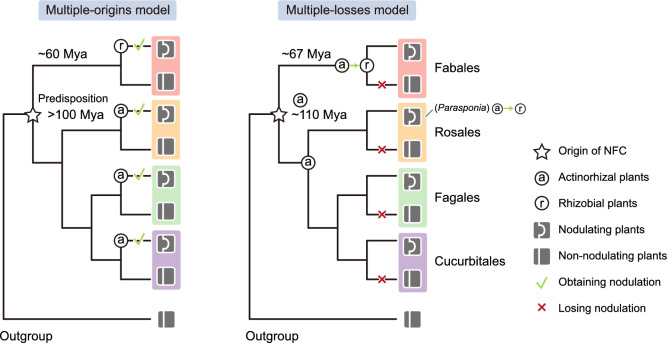
Two main hypotheses for the origin of NFC. The multiple-origin hypothesis suggests that a predisposition for nodulation evolved in the origin of the NFC over 100 Mya. Subsequently, around 60 Mya, independent acquisitions of nodule formation (indicated by green tick marks) occurred (Soltis et al. 1995; Werner et al. 2014). The circled letters ‘a’ and ‘r’ denote the acquisition of nodulation with *Frankia* and *Rhizobium*, respectively. The multiple-losses hypothesis proposes that a single acquisition of nodulation with *Frankia* took place at the origin of the NFC around 110 Mya. Following this, multiple losses of nodulation occurred in the non-nodulating lineages (indicated by red crosses). Two independent switches of symbionts from *Frankia* to rhizobia occurred: once in the ancestor of the legumes within the Fabales around 67 Mya, and once in the *Parasponia* genus within the Rosales (Griesmann et al. 2018; van Velzen et al. 2018)

Notably, the formation of nodules is a rather rare trait, as plants belonging to only 10 out of the 28 NFC plant families can form nodules (Doyle 2011; Soltis et al. 1995). This observation has led to two primary hypotheses regarding the origin of the NFC: One proposing a multiple-origins model, where nodulation evolved independently within the NFC on multiple occasions; the other advocating for a single origin of nodulation in the NFC, followed by multiple losses of this trait in different now non-nodulating species (Fig. 2).

The multiple-origins hypothesis was initially widely accepted and is supported by the notable variation observed in nodule ontogeny and physiology among nodulating lineages (Ott et al. 2005; Pawlowski and Demchenko 2012). A phylogenetic study conducted in 1995 analyzed the sequences of the chloroplast gene *RbcL* encoding the large subunit of Rubisco from 499 plant species, revealing that all families within the NFC share a common ancestor, suggesting a single evolutionary origin for the capacity to engage in a symbiotic relationship (Soltis et al. 1995). Thus, a two-step hypothesis was proposed, whereby the ancestor of the NFC acquired a novel predisposition that would ultimately permit the evolution of nodulation. Subsequent studies, particularly a large phylogenetic analysis of the genome sequences of seven loci from 3,467 angiosperm species, reached similar conclusions (Doyle 2011; Werner et al. 2014). These studies support the idea that the members of the NFC shared a common ancestor approximately 100 Mya, in which the predisposition for nodulation newly arose, with six to ten independent acquisitions or convergent evolution steps toward nodulation subsequently arising in different lineages of the NFC.

However, despite decades of research, the exact nature of this hypothetical predisposition for nodulation has remained elusive. Thus, the hypothesis of a single gain of nodulation has been suggested as an alternative, proposing that the common ancestor of the NFC acquired the capacity for nodulation rather than a predisposition towards it, followed by multiple losses of this trait (van Velzen et al. 2019). In a genome-wide comparative analysis of 37 plant species, Griesmann et al. identified signatures of multiple independent loss-of-function events for several essential nodulation-related genes (Griesmann et al. 2018). In particular, loss of function of *NODULE INCEPTION* (*NIN*), encoding a transcription factor crucial and specific for symbiotic nodulation (Schauser et al. 1999), was observed in 10 out of 13 non-nodulating species within the NFC, providing strong evidence for the multiple-losses model. Recently, the same group conducted a larger-scale phylogenomic and phylotranscriptomic analysis across 88 species, lending further support to their initial conclusions (Zhang et al. 2024c).

Furthermore, phylogenetic studies that support the multiple-losses hypothesis have suggested that nodulation first evolved with *Frankia* species rather than rhizobia, followed by one or more evolutionary transitions (van Velzen et al. 2018). The Fabaceae family experienced multiple WGDs, including from their most recent common ancestor (MRCA) (Cannon et al. 2015; Koenen et al. 2021). These WGDs may have increased the copy number of nodulation-related genes, facilitating the transition from actinorhizal to rhizobial nodulation. To investigate this hypothesis, Zhao et al. compiled the genomes of 463 Fabaceae species across 333 genera and reconstructed a refined phylogeny (Zhao et al. 2021). Phylogenetic analysis of 30 gene families that include genes with established roles in SNF, such as the *NIN-LIKE PROTEIN* (*NLP*) family to which *NIN* belongs, supported the multiple-losses model within Fabaceae. Furthermore, these authors identified several nodulation-related genes that underwent a duplication in the MRCA of Fabaceae, suggesting that the shift to rhizobial nodulation likely occurred at this ancestral point or earlier. Collectively, this phylogenomic inquiry supports the notion that the evolution of nodulation in Fabaceae involved several transitions from actinorhizal to rhizobial nodulation, followed by multiple losses.

Clearly, both theories have their individual merits and limitations when trying to explain how the NFC arose, and it is conceivable that these two scenarios might coexist (Parniske 2018). It is worth noting that the number of assembled genomes for NFC plant species remains limited, particularly those of high quality (Table S1). It is indeed challenging to assemble the genomes of non-model plant species, for which homozygous materials are often lacking. Nevertheless, the advent of long-read sequencing technology presents a promising solution to this problem (Zhou et al. 2020). With the emergence of more high-quality genomes, scientists may finally uncover the origin of the NFC and decipher the distinctive traits of the nodulating plants. Ideally, the genomes of all plants from the NFC should be assembled, empowering comparative analyses to help identify shared nodulation-specific genes among nodulating plants, which may reveal the identity of genes responsible for the predisposition towards nodulation. Furthermore, such analyses would allow insights into whether non-nodulating plants have lost one or more essential nodulation-specific genes, thereby validating the multiple-losses hypothesis.

## Profiling the transcriptome during symbiotic nodulation

Transcriptome studies explore the overall status of transcript levels, serving as a vital tool for uncovering molecular events, regulatory mechanisms, and functional genes. Before the genomes of model legumes were sequenced and assembled, researchers had to resort to sets of complementary DNAs (cDNAs) cloned as expressed sequence tags (ESTs) from specific tissues. These ESTs were spotted onto slides to create DNA arrays for transcriptome profiling (Duggan et al. 1999), enabling the identification of nodulin genes across various legumes.

In 2002, Colebatch et al. developed an array containing probes for 1,500 *L. japonicus* genes, revealing 83 genes that were more highly expressed in nodules than in roots (Colebatch et al. 2002). Subsequent research developed an array containing 52,749 probe sets for *L. japonicus*, and gathered samples from a range of organs, including nodules at various developmental stages, to identify nodulin genes expressed at each stage (Høgslund et al. 2009). Leveraging this result, the researchers discovered that the expression of ethylene biosynthesis genes was downregulated 1 day after rhizobium inoculation, consistent with previous findings (Nukui et al. 2000; Oldroyd et al. 2001). However, this pattern was reversed by 7 days post-inoculation, highlighting the complex regulation of this phytohormone on nodule development. Building on this foundation, the *L. japonicus* Gene Expression Atlas (LjGEA) database was established, providing a valuable resource for studying the changes in gene expression associated with nodulation progression (Verdier et al. 2013).

For analysis in *M. truncatula*, Benedito et al. utilized the Affymetrix GeneChip Medicago Genome Array, which contains 50,900 sets of probes covering most *M. truncatula* genes, to investigate gene expression patterns in key tissues including nodules (Benedito et al. 2008). This comprehensive dataset revealed the intricate expression profiles of over 26,000 genes during nodule development, with 30.2% of genes showing elevated expression levels in nodules compared with roots. Among these, 532 putative transcription factor genes were identified, providing insights into the regulatory networks governing nodulation. By integrating this information with other transcriptome datasets, researchers created the *Medicago truncatula* Gene Expression Atlas (MtGEA), another valuable resource for studying nodulation (He et al. 2009).

With the advancement of sequencing technologies, transcriptome sequencing analysis with short sequencing reads, known as RNA-seq, has emerged as a powerful tool for investigating SNF. RNA-seq can determine gene expression levels without a reference genome. Moreover, RNA-seq offers heightened sensitivity and a broader range for detecting gene expression compared with array-based methodologies (Nagalakshmi et al. 2008), thereby enabling more precise analyses, such as correlation analysis, co-expression analysis, and gene regulatory network analysis (Movahedi et al. 2012; Singh et al. 2022; Stark et al. 2019). For instance, Severin et al. employed RNA-seq to analyze the transcriptomes of various soybean tissues, discovering that the expression profile of nodules is more similar to that in roots than to that in other tissues (Severin et al. 2010), providing molecular support for the observation that nodules typically arise from root cortical cells (Brewin 1991). In 2013, Boscari et al. investigated *M. truncatula* nodule development, generating an impressive 5 Gb data (representing a tenfold coverage of the *M. truncatula* genome). They uncovered 7595 previously unidentified transcribed regions linked to 5264 annotated genes, significantly enhancing the annotation of the *M. truncatula* genome and illustrating the transcriptional reprogramming that goes on during nodulation (Boscari et al. 2013). A recent transcriptome analysis in *M. truncatula* revealed that most changes in gene expression during lateral root initiation also occur during nodule organogenesis (Schiessl et al. 2019). These findings collectively suggest that nodulation is likely achieved through the reprogramming of the root transcriptome, and that identifying related genes can offer a greater understanding of the origin and essential conditions of nodulation.

Furthermore, full-length transcripts can be assembled from RNA-seq reads, thereby enabling the identification of alternative splicing (AS), a prevalent phenomenon in eukaryotic genes that significantly enhances transcriptome and proteome diversity. In 2015, a study was conducted to explore AS during nodulation by assembling full-length transcripts from RNA-seq datasets derived from various tissues, including nodules, of *Pisum sativum* (Alves-Carvalho et al. 2015). Nevertheless, due to limitations in read length at the time, and despite discovering many genes potentially undergoing AS, the researchers could only definitively uncover AS in a few genes. A recent study leveraged long-read sequencing to overcome this issue and investigated the full-length transcriptome of underground tissues from soybean, both inoculated and uninoculated, uncovering 7,874 differential splicing events during nodule development (Liu et al. 2022). Notably, 2,008 genes appeared to be transcribed as multiple RNA isoforms in a stage-specific manner, or switching from one major transcript isoform to another following rhizobium inoculation, suggesting that AS plays a crucial role in regulating nodulation.

Since the groundbreaking discovery of microRNAs (miRNAs) in 1993, the scientific community has been intrigued by their regulatory roles (Lee et al. 1993; Pasquinelli et al. 2000; Wightman et al. 1993). Accordingly, the influence of miRNAs on nodulation has been investigated. Subramanian et al. successfully isolated and sequenced small RNAs from both the inoculated and naïve roots of soybean plants, identifying 55 miRNA families, the abundance of many being specifically upregulated during infection (Subramanian et al. 2008). Further research by Li et al. demonstrated that transgenic overexpression of the precursors for certain miRNAs, including pri-*miR482*, pri-*miR1512*, and pri-*miR1515*, significantly enhances nodule formation, confirming the regulatory effects of miRNAs on nodulation (Li et al. 2010).

Long non-coding RNAs (lncRNAs) also represent a critical regulatory layer of gene activity (Nagano and Fraser 2011; Ponting et al. 2009), and efforts have been made to study their function in nodulation. Traubenik et al. combined translating ribosome affinity purification (TRAP) with RNA-seq to explore the mechanisms of translational regulation of mRNAs during the initial phase of infection in *M. truncatula* roots (Traubenik et al. 2020). Their findings indicated that a short variant of the lncRNA *Trans-acting small interference RNA 3* (*TAS3*) likely regulates nodule formation and rhizobial infection through auxin signaling, emphasizing the involvement of lncRNAs in regulating symbiotic interactions.

## Defining spatiotemporal transcriptomes

While whole-organ transcriptome studies of nodule development provide valuable insights into nodulation, it is essential to recognize that nodules comprise diverse cell layers and cell types. A precise analysis of gene expression within specific tissues or cells is therefore necessary for a deeper understanding of nodulation. In particular, the infection zone, a unique tissue characterized by infected cells containing symbiosomes, necessitates a thorough examination to decipher the establishment of symbiosis.

The laser-capture microdissection (LCM) technique is highly effective for isolating various tissue types across different plant species (Brewin 1991; Vasse et al. 1990), making it ideal for investigating the development of indeterminate nodules, such as those in *M. truncatula*. These nodules possess a persistent meristem that generates a continuous gradient of tissues undergoing differentiation, thus representing successive developmental stages (Vasse et al. 1990; Zhang et al. 2024b). Roux et al. combined LCM with RNA-seq to analyze *M. truncatula* nodules, identifying five regions corresponding to sequential developmental stages of the infection zone (Roux et al. 2014). This study revealed hundreds of candidate functional genes at various stages. Notably, the authors discovered that several transcription factor genes pivotal for controlling the root apical meristem were also expressed in the nodule meristem, consistent with the notion that nodulation is likely achieved through a transcriptomic reprogramming of the root. Among these transcription factor genes, *SCARECROW* (*SCR*) was confirmed to be crucial for nodule primordium formation, as evidenced by a subsequent study (Dong et al. 2021).

The advent of scRNA-seq has enabled a single-cell resolution analysis of transcriptomes. Ye et al. utilized this technology to profile the transcriptome of individual cells within *M. truncatula* nodules, identifying 13 distinct cell clusters (Ye et al. 2022). Their analysis demonstrated that uninfected cells within the nitrogen fixation zone actively participate in nitrogen assimilation by engaging in asparagine biosynthesis. The following year, two studies employed scRNA-seq to investigate single root cells in *M. truncatula* during early infection stages, uncovering numerous previously unknown candidate genes whose expression is responsive to infection (Cervantes-Pérez et al. 2023; Liu et al. 2023b). Particularly, a gene co-expression analysis revealed the similar expression pattern of *MtFERONIA* (*MtFER*) and *LysM-RLK 3* (*MtLYK3*), the latter being the ortholog of *LjNFR1* (Liu et al. 2023b). Further studies demonstrated that MtFER is phosphorylated by MtLYK3 and plays a role in rhizobial symbiosis, thereby confirming the participation of this new factor in plant responses to symbiotic signals.

ScRNA-seq has also been applied to determinate nodules, characterized by the loss of meristems upon maturity (Cervantes-Pérez et al. 2024; Sun et al. 2023; Wang et al. 2022). While these studies successfully identified various cell types, they were limited to a single developmental stage due to the lack of successive differentiated tissues within determinate nodules (Ferguson et al. 2010; Szczyglowski et al. 1998). Additionally, discerning the developmental states of determinate nodules based solely on visual appearance is challenging, compounded by difficulties in tracking their developmental timelines underground.

The emergence of spatial transcriptomics presents a promising solution to this sampling challenge by producing in situ transcriptomes, which facilitates visualization of transcriptome for individual samples throughout nodule development (Chen et al. 2022; Ståhl et al. 2016). Recently, our group applied spatial transcriptomics to explore the development of determinate nodules in *L. japonicus* (Ye et al. 2024). Our findings complemented the above-mentioned notion that nodules exhibit similarities to roots (Severin et al. 2010), by illustrating how nodule peripheral tissues resemble root tissues, while the nodule infection zone represents a distinct feature. Additionally, we delineated the developmental trajectories of both the infection zone and peripheral tissues, unraveling the intricate progression and functional gene sets necessary for achieving symbiosis and material exchange between host and rhizobia.

Although spatial transcriptomics offers location information, it currently faces a low-resolution challenge, together with limited sequencing depth. By contrast, single-cell data provide higher resolution and depth. By merging these two methods, researchers can create a high-quality single-cell spatial transcriptome (Longo et al. 2021; Moncada et al. 2020; Saviano et al. 2020). Liu et al. employed such a combined approach to study soybean nodules, capturing transitional states during their maturation (Liu et al. 2023a). In tropical legumes like soybean, ureides are the primary form of nitrogen export from nodules (Collier and Tegeder 2012). The study by Liu et al. (2023a) revealed that distinct uninfected cell types are responsible for ureide biogenesis and transport, uncovering an intricate compartmentalization within uninfected cells during nodulation.

Another limitation of current spatial transcriptomics methods lies in their dependence on capturing mRNA through polyA tails, which hinders the detection of RNA species devoid of such polyA tails, including mature miRNAs and certain lncRNAs. Developing a capturing strategy to detect these RNA types would allow for a more comprehensive spatiotemporal transcription atlas to be created to gain insights into nodule development. Notably, for SNF, such a method would offer an additional practical application by detecting the transcriptome of rhizobia, which also lack polyA tails. The integration of transcriptome data from both participants would yield greater insights into their symbiotic interaction. At a simpler scale, several bulk transcriptomic analyses have been conducted on both hosts and symbionts (Lang and Long 2015; Sauviac et al. 2022; Zhang et al. 2023). For example, a comparative transcriptome analysis of wild-type plants and a *nodules with activated defense 1–1* (*nad1-1*) mutant lacking nodulation identified differentially expressed genes in both *M. truncatula* and its symbiont *Sinorhizobium meliloti*, demonstrating that the host employs plant immunity to control substantial bacterial colonization within nodules (Zhang et al. 2023).

## Revealing epigenetic modifications during nodulation

Epigenetic modifications represent an important regulatory system that enables heritable changes in gene expression; types of modification include chemical modifications such as methylation and acetylation of DNA and its associated histones, as well as changes in DNA accessibility and chromatin conformation (Waddington 2012). A study conducted in 2016 utilized bisulfite sequencing coupled with genomic capture to pinpoint 474 differentially methylated regions (DMRs) during nodule development in *M. truncatula* (Satgé et al. 2016). This study determined that the demethylase DEMETER (DME) plays a crucial role in differential methylation, with lower *MtDME* expression levels resulting in the hypermethylation of 278 DMRs and substantial defects in nodule differentiation.

The extent of DNA accessibility and histone modifications during nodulation have also been explored. The growth of nodule cells in legumes is driven by consecutive cycles of endoreduplication (Vinardell et al. 2003). A study conducted in 2017 revealed a correlation between ploidy levels and transcriptional reprogramming (Nagymihály et al. 2017). By performing the transposase-accessible chromatin sequencing (ATAC-seq) technique on nodules at various developmental stages, they demonstrated that changes in chromatin accessibility were linked to the observed transcriptional alterations. Furthermore, chromatin immunoprecipitation (ChIP) assays showed that histone modifications, particularly the levels of H3K27me3 and H3K9ac, were correlated with the expression of several genes. Another study employed ChIP followed by sequencing (ChIP-seq) and RNA-seq to investigate mature nodules in soybean, uncovering a positive correlation between changes in H3K4me3 levels and the transcript levels of functional genes within the nodules (Wang et al. 2020). These studies collectively illustrate the critical role of epigenetic modifications in nodulation.

Similar to transcriptome analysis, several high-resolution epigenomic techniques have been devised, and these hold promise for investigating nodulation (Luo et al. 2020; Preissl et al. 2023). In 2022, Pecrix et al. integrated LCM with whole-genome bisulfite sequencing (WGBS) to investigate DNA methylation during *M. truncatula* nodule development (Pecrix et al. 2022), enhancing their previous research that had focused on a WGBS analysis of whole nodules (Satgé et al. 2016). Their findings revealed significant dynamics in DNA methylation during the later stages of nodule development. Notably, they detected an increase in methylation in the CHH context (with C being cytosine and H being any nucleotide except guanine) in the differentiation and fixation zones across all chromosomes, while they observed minimal variation when looking at whole nodules, emphasizing the advantages of detailed epigenomic data. Recently, a study constructed a spatially resolved single-cell atlas of gene expression, coupled with chromatin accessibility, across ten soybean tissues, identifying 103 distinct cell types and thousands of enriched motifs potentially bound by transcription factors (Zhang et al. 2024a). Among these motifs were four motifs for known master regulators of nodulation, such as GmNIN, along with two unknown *cis*-regulatory motifs specific to infected cells, which may potentially play crucial roles in SNF.

## Applying proteomics analyses to SNF

Proteomics, being the high-throughput examination of protein abundance and modifications, has emerged as a crucial tool in biological research. It reveals additional layers of post-transcriptional regulation, including protein modifications and degradation, thereby offering complementary insights to genomics and transcriptomics (Depuydt et al. 2023; Vogel and Marcotte 2012; Zhu et al. 2003). In 2014, Dam et al. utilized proteomics to investigate nodule development in *L. japonicus*, comparing the composition of proteins in nodules at two critical stages: pre-nitrogen fixation (white) and mature nitrogen-fixing (red) nodules (Dam et al. 2014). Using 2D gel electrophoresis, they identified 780 and 790 protein spots in nodules at each of these developmental stages. White nodules accumulated higher levels of pathogen-related proteins, heat shock proteins (HSPs), and redox-active proteins, indicating elevated stress during this phase. By contrast, red nodules were enriched in nodulins such as leghemoglobin, asparagine synthetase, and glutamine synthetase, reflecting their role in nitrogen fixation.

In 2016, a comprehensive proteomic study of *M. truncatula* examined the proteomes of seven major organs, including nodules across three developmental stages (Marx et al. 2016). By integrating transcriptomic and proteomic data, researchers unveiled a previously unknown symbiosis-specific regulatory network, elucidating co-expression and co-regulatory patterns. They also quantified post-translational modifications (PTMs) essential for protein function, identifying 26,000 phosphorylation sites and 600 acetylation sites. Importantly, they highlighted nodule-specific phosphorylation events, such as the phosphorylation of Does not Make Infections 1 (MtDMI1), a crucial calcium channel involved in symbiotic signaling, providing a valuable dataset for investigating PTMs governing nodulation.

In addition to whole-organ exploration, proteomic studies have also focused on the symbiosome, given the pivotal role of this unique organelle (Coba de la Peña et al. 2017; Liu et al. 2018; Zhang et al. 2024b). Wienkoop and Saalbach employed a high-throughput proteomic approach to investigate the symbiosome membrane in *L. japonicus*, identifying approximately 94 proteins, many more than previous reports (Panter et al. 2000; Saalbach et al. 2002; Wienkoop and Saalbach 2003). This finding underscores the complexity of the symbiosome membrane, revealing not only well-documented transporters essential for material exchange (Banasiak et al. 2021; Li et al. 2024a), but also vesicle-associated proteins, such as GTP-binding proteins and vesicle receptors, and signaling proteins such as receptor kinases and calmodulin. This study supports the notion that the symbiosome is a vesicle that shares similarities with cellular invasion by pathogens during phagocytosis (Jones et al. 2007).

The availability of genome sequences for model legumes has enhanced proteomic investigations into symbiosomes. A study on soybean identified 197 proteins as constituents of the symbiosome membrane, complementing the list of known symbiosome components (Clarke et al. 2015). Notably, this research also highlighted multiple vesicle-related proteins as being localized to the symbiosome membrane, including components of the soluble N-ethylmaleimide-sensitive factor attachment protein receptor (SNARE) complex and several small G proteins, which are crucial for vesicle fusion, emphasizing the vesicle-like nature of the symbiosome.

**Fig. 3:**
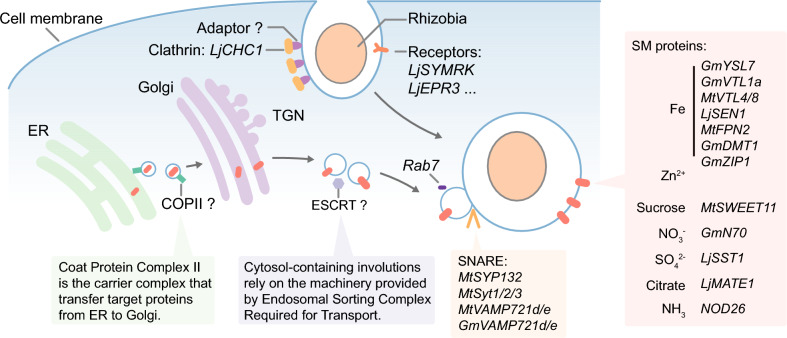
Vesicle trafficking mechanism involved in symbiosome development. Significant progress has been made in identifying functional proteins on the symbiosome membrane, including several transporters that facilitate essential material exchange (Brear et al. 2020; Escudero et al. 2019; Gavrin et al. 2021; Hakoyama et al. 2012; Hwang et al. 2010; Kaiser et al. 2003; Krusell et al. 2005; Kryvoruchko et al. 2016; Liu et al. 2020; Moreau et al. 2002; Suganuma et al. 2003; Takanashi et al. 2013; Vincill et al. 2005; Walton et al. 2020; Wu et al. 2023). However, understanding the formation and maturation of symbiosome remains incomplete. During symbiosome formation, in addition to well-characterized signal receptors like LjSYMRK and LjEPR3 (Kawaharada et al. 2015; Stracke et al. 2002), studies have indicated that clathrin is also involved, suggesting that the symbiosome may form through a clathrin-mediated endocytosis (CME) pathway (Wang et al. 2015). During maturation, proteins like Rab7 and several belonging to the SNARE complexes have been identified as participants in vesicle fusion processes (Catalano et al. 2007; Cheon et al. 1993; Gavrin et al. 2016, 2017; Ivanov et al. 2012; Limpens et al. 2009; Pan et al. 2016). Yet, research on additional proteins involved in modification and transport, such as those in the COPII and ESCRT complexes, is still limited (Aniento et al. 2022)

Despite progress in our understanding of the vesicle trafficking mechanisms underlying symbiosome development (Ivanov et al. 2012; Limpens et al. 2009; Pan et al. 2016), there is still much to uncover (Aniento et al. 2022) (Fig. 3). For example, scientists have discovered that proteins belonging to the vesicle-associated membrane protein 72 (VAMP72) family, which are essential components of the SNARE complex, participate in symbiosome development in both alfalfa (*Medicago sativa*) and soybean (Gavrin et al. 2016; Ivanov et al. 2012). Furthermore, the recognition of vesicles and their binding to the SNARE complex often requires the assistance of small G proteins, such as Rab7 (Langemeyer et al. 2018; Mizuno-Yamasaki et al. 2012). Lower expression levels of *Rab7* are associated with abnormal symbiosome development in *M. truncatula* and *Vigna aconitifolia* (Cheon et al. 1993; Limpens et al. 2009). Notably, when genes encoding proteins located in symbiosomes are heterologously expressed in non-symbiotic nitrogen-fixing plants, their encoding proteins tend to localize to vacuoles. Mutations of several vesicle trafficking–related genes that affect symbiosome development also result in abnormal vacuole development, suggesting potential common pathways in the development of these two organelles (Brear et al. 2020; Gavrin et al. 2021; Meckfessel et al. 2012; Wang et al. 2010). In investigations of vacuole biogenesis in the model plant *Arabidopsis thaliana*, researchers have delineated two major vesicle fusion pathways: one mediated by Rab7 and VAMP71, and the other coordinated by Rab5 and VAMP72 (Takemoto et al. 2018). However, *Rab5* is not expressed in *M. truncatula* nodules (Limpens et al. 2009), suggesting that symbiosome development may be jointly regulated by Rab7 and VAMP72, highlighting differences between symbiosome and vacuole development. Comparative proteomic studies of these two organelles would help unravel the underlying mechanisms.

The recent discovery of the nitrogen-fixing organelle *Candidatus* Atelocyanobacterium thalassa (UCYN-A) within the marine alga *Braarudosphaera bigelowii* offers promising prospects for the potential transfer of the symbiosome to other plants (Coale et al. 2024; Cornejo-Castillo et al. 2024). In their study, Coale et al. utilized proteomics to assess how many of the proteins present in UCYN-A are encoded by the *B. bigelowii* genome. They discovered that 28% of the UCYN-A proteome proteins during the daytime and 11% during the nighttime are encoded by the *B. bigelowii* genome. Furthermore, alignment of the proteins encoded by the *B. bigelowii* genome that are detected in UCYN-A revealed a series of conserved motifs within a C-terminal region. Notably, proteomics analysis failed to detect peptides matching this region, suggesting that it may be cleaved or not translated, potentially identifying it as the transit peptide that directs proteins to UCYN-A. These proteomic analyses were pivotal in establishing UCYN-A as an organelle in *B. bigelowii* cells.

Proteomics also help unveil interactions between proteins involved in nodulation (Wu et al. 2021). A co-immunoprecipitation-based proteomics screen identified proteins associated with LjNFR5 (Wong et al. 2019), leading to the identification of a receptor-like cytoplasmic kinase, termed NFR5-interacting cytoplasmic kinase 4 (NiCK4). Further investigation demonstrated that NiCK4 is a component of the Nod factor signaling pathway downstream of LjNFR5, thereby expanding the list of components involved in symbiotic signal reception.

Another application of proteomics is in deciphering signaling initiated by small peptides, some of which orchestrate plant growth and development (Durgo et al. 2015; Patel et al. 2018; Zhang et al. 2006). Such investigations have been extended to nodulation (Matsubayashi 2014; Tan et al. 2024). Patel et al. identified 759 spectra corresponding to the secreted products of 12 peptide hormones, including four C-terminally encoded peptides (CEPs), two CLAVATA3 (CLV3)/ESR-related peptides (CLEs), and six xylem sap-associated peptides (XAPs) (Patel et al. 2018). Their findings demonstrated that CEPs can enhance the number of root nodules, a previously reported phenomenon (Imin et al. 2013). Notably, Patel et al. showed that this effect was enhanced through hydroxylation modification of specific proline residues in the CEPs, offering insights into the role of peptide modification in nodulation.

## Conclusions

SNF is fascinating and valuable, but it is also highly complex. While around 200 genes have been identified as essential for SNF (Mergaert et al. 2020; Roy et al. 2020), transcriptomic studies indicate that thousands more may play roles that have yet to be explored (Benedito et al. 2008; Høgslund et al. 2009). Moreover, the proteins encoded by some of these genes may cooperate to carry out functions, while others may be functionally redundant, adding to the complexity of SNF research. A comprehensive analysis of SNF from various perspectives is essential, and high-throughput omics approaches are especially effective for this endeavor. Over the years, researchers have generated large multi-omics datasets, yielding critical discoveries, but the high-dimensional nature of these datasets is ripe for deeper exploration.

Multi-omics analysis is an effective strategy for uncovering this hidden information. By integrating multiple datasets from distinct omics sources, we can gain insights into SNF from diverse angles, effectively lowering the noise associated with single-dimensional studies and enhancing the reliability of the resulting findings. One such endeavor is the work by Colebatch et al., who combined transcriptomics and metabolomics to investigate *L. japonicus* nodule development (Colebatch et al. 2004). Their metabolomic analysis revealed a distinct metabolic profile in nodules, which reflected the global metabolic changes inferred from transcriptome data, providing an unprecedented overview of metabolic differentiation during nodule development.

Recently, the advancement of artificial intelligence (AI) has introduced powerful tools for analyzing omics data. Since the introduction of AlphaFold from Google DeepMind in 2018, the potential for AI in biology has garnered widespread attention (Senior et al. 2020). Researchers are now focusing on developing AI tools to analyze large datasets, potentially advancing SNF research. For example, transcriptomic reprogramming during nodulation often stems from variation in promoter sequences, which traditional alignment methods struggle to analyze due to excessive sequence diversity in the promoter region (de Boer and Taipale 2024). Notably, recent AI tools have made impressive progress in addressing this challenge (Dudnyk et al. 2024; Hao et al. 2024; Kaplow et al. 2023). If similar tools tailored to nitrogen-fixing plants can be developed, they will greatly enhance our understanding of the transcription networks underlying SNF.

AI has also revitalized interest in phenomics, an effective methodology for identifying functional genes. Previously, phenomics was hindered by labor-intensive phenotypic data acquisition (Houle et al. 2010). Advances in AI-driven recognition technologies now make large-scale phenotypic data collection feasible (Yang et al. 2020), potentially accelerating SNF studies. Several mutant libraries of model legumes have already been established, providing valuable resources for phenomics research (Bolon et al. 2011; Penmetsa and Cook 2000; Perry et al. 2003; Szczyglowski et al. 1998). Furthermore, with the maturity of genome-editing tools such as clustered regularly interspaced short palindromic repeats (CRISPR)/CRISPR associated nuclease (Cas) systems (Gao et al. 2024; Jinek et al. 2012; Wang and Doudna 2023), researchers can now create targeted mutants previously unattainable by traditional methods, as exemplified by the isolation of a single plant harboring mutations in three tandemly located leghemoglobin genes in *L. japonicus* (Wang et al. 2019).

In addition, research on single-cell and spatial transcriptomics has highlighted cell specificity in nodules. Recently, Dolatmoradi et al. have developed a high-throughput single-cell metabolomics platform to study soybean nodules, discovering the metabolic heterogeneity of infected cells, which is defined by the presence of proliferating bacteria in differentiating infected cells and quiescent bacteria in fully functional cells (Dolatmoradi et al. 2023). More single-cell omics studies, including epigenomics as mentioned above, are essential for delving deeper into SNF (Hu et al. 2023; Labib and Kelley 2020; Perkel 2021).

Although significant progress has been made, much about SNF remains to be discovered. Omics technologies have demonstrated their ability to address this complex biological phenomenon. As omics approaches continue to evolve, they hold great promise for advancing our understanding of SNF and facilitating the development of new nitrogen-fixing crop varieties.

## Supplementary Information

Below is the link to the electronic supplementary material.

Supplementary file1 (XLSX 12 KB)

## Data Availability

Data sharing is not applicable to this article as no datasets were generated or analyzed during the current study.
